# Financial Toxicity and Coronary Heart Disease: A Dual-Level Cross-Sectional and Spatial Analysis of Adults in the United States, 2022-2024

**DOI:** 10.7759/cureus.112164

**Published:** 2026-07-06

**Authors:** Carlos Acosta-Batista, Karina Verrier Maden, Rosemarie Morera Gomez, Andrew A López Sánchez, Laura B Diaz Perez, Leonardo A Almaguer Gongora

**Affiliations:** 1 Clinical Research, Primary Care - Research Initiative (PCRI), Miami Lakes, USA; 2 Medicine, University of Louisville Physicians, Louisville, USA; 3 Internal Medicine, Asociación Española, Montevideo, URY; 4 Family Medicine, Included Health, Lewiston, USA; 5 Internal Medicine, HCA Florida Aventura Hospital, Aventura, USA

**Keywords:** bayesian hierarchical modeling, behavioral risk factor surveillance system, coronary heart disease, family medicine, financial toxicity, health disparities, spatial epidemiology

## Abstract

Introduction

Coronary heart disease (CHD) remains a leading cause of mortality in the United States. While traditional risk factors are well established, the structural impact of financial toxicity, defined as the inability to afford medical care, on cardiovascular outcomes is undercharacterized in nationally representative populations.

Methods

A dual-level analysis was conducted. At the individual level, a pooled cross-sectional analysis using 2022-2024 Behavioral Risk Factor Surveillance System (BRFSS) data (N=1,336,111) was performed to calculate prevalence ORs. At the macro level, a Bayesian spatial hierarchical model using the Besag-York-Mollié (BYM) specification was implemented to evaluate the geographic association between state-level financial toxicity and CHD mortality.

Results

Individual-level analysis revealed that financial toxicity was strongly associated with premature CHD, corresponding to more than three-fold higher odds of CHD among adults aged 25-44 years (OR=3.38; 95% CI: 2.78-4.12). At the macro level, structural financial toxicity and state uninsured rates were highly collinear (r=0.88). State-level multivariable modeling (R²=0.42) identified age-adjusted CHD prevalence as the dominant predictor of mortality. Furthermore, Bayesian spatial modeling (BYM) confirmed financial toxicity as a credible, independent correlate of cardiovascular mortality (β=2.51; 94% highest-density interval (HDI): 0.65-4.39), with pronounced residual geographic risk clustering in the Midwest and Mid-Atlantic regions.

Conclusions

Financial toxicity is a critical structural correlate of premature cardiovascular disease and regional mortality. Improving population-level cardiovascular outcomes requires integrating structural competence and financial screening into primary care, alongside addressing state-level healthcare policy gaps.

## Introduction

Coronary heart disease (CHD) continues to be a major public health challenge and remains one of the leading causes of death in the United States. The magnitude of this public health crisis is substantial; in 2020, one in three US adults received care for a cardiovascular risk factor or condition [1]. The economic burden is equally severe, with cardiovascular disease and stroke accounting for $251 billion in direct healthcare spending and $156 billion in lost productivity in 2019 [1]. While substantial progress has been made in identifying traditional cardiovascular risk factors, increasing attention has been directed toward the role of social and economic conditions in shaping cardiovascular health. Among these factors, financial toxicity has emerged as a growing concern, particularly as rising healthcare costs place considerable strain on individuals and families [2].

Financial toxicity extends beyond direct medical expenses and encompasses the broader psychological and behavioral consequences of financial hardship, including medical debt, psychological distress, delayed care, and medication nonadherence [3]. The scope of this issue is expanding; in a US poll from 2022, 38% of patients reported that they put off treatment due to costs, representing a nearly 50% increase from 2021 [3]. Furthermore, medication nonadherence rates among patients with heart failure are estimated to be between 29% and 63% [4]. Economic strain is linked to cardiovascular health through multiple pathways. Individuals experiencing financial difficulties may delay seeking medical attention, skip preventive services, reduce medication adherence, or face challenges maintaining recommended lifestyle modifications. In addition, persistent financial concerns can function as a chronic stressor, potentially contributing to physiological changes associated with cardiovascular disease development. Evidence suggests that prolonged exposure to stress may trigger neuroendocrine and inflammatory responses, which are correlated with increased blood pressure, metabolic dysregulation, and vascular dysfunction, all of which are recognized contributors to cardiovascular disease [2].

Recent literature has strengthened the link between socioeconomic adversity and cardiovascular outcomes. Lower socioeconomic status has been associated with higher rates of cardiovascular events and mortality, even among individuals who maintain healthier lifestyles [5]. Furthermore, financial barriers to healthcare are correlated with poorer recovery and long-term outcomes among patients with cardiovascular conditions [6]. These barriers are driven by substantial out-of-pocket (OOP) expenditures; for instance, the estimated total direct OOP costs of heart failure appear to exceed $4,000 annually [3]. This creates a severe financial burden, as an estimated one in seven families with a member with heart failure spends more than one-fifth of their postsubsistence income on medical expenses [3]. Financial stress is associated with an elevated risk of adverse cardiovascular outcomes, while longitudinal studies suggest a strong directional relationship between chronic financial stress and cardiovascular disease risk [7].

Although financial stress has been increasingly recognized as a correlate of cardiovascular health, the relationship between healthcare affordability and CHD prevalence has not been fully characterized in nationally representative populations [8]. Therefore, this study seeks to examine the association between financial toxicity, specifically operationalized in this analysis as the inability to see a doctor in the past 12 months due to cost, and the prevalence of CHD in the United States. A better understanding of this relationship may provide valuable insights into the broader implications of healthcare affordability for cardiovascular health and help guide future public health and policy interventions.

## Materials and methods

Study design and data sources

This study utilized a dual-level analytical approach, combining an individual-level cross-sectional design with a macro-level spatial ecological analysis to evaluate financial toxicity as a structural correlate of CHD. These analyses were conducted as complementary but distinct parallel analyses, rather than as a single multilevel model linking individual respondents to state-level covariates, to reduce the risk of cross-level ecological fallacies.

Individual-level data on CHD prevalence, financial toxicity, and sociodemographic variables were extracted from the CDC Behavioral Risk Factor Surveillance System (BRFSS) for the years 2022, 2023, and 2024 [9,10]. The study period spanned from January 1, 2022, to December 31, 2024. To appropriately account for the complex survey design across the pooled 36-month dataset, annual BRFSS final weights (_LLCPWT) were rescaled by dividing by three. Stratification (_STSTR) and primary sampling unit (_PSU) variables were incorporated to ensure valid variance estimation for all prevalence and confidence interval calculations.

At the ecological state level (n=50 U.S. states, including Alaska and Hawaii), data were aggregated and merged with two additional national databases:

CDC WONDER (Underlying Cause of Death)

State-level age-adjusted mortality rates for ischemic heart disease (ICD-10: I20-I25) per 100,000 population were extracted and averaged across the 2022-2024 period [11].

Kaiser Family Foundation (KFF)

State-level uninsured rates, averaged across 2022-2024, and Medicaid expansion status were extracted [12].

The study population included all adult respondents aged ≥18 years from the pooled BRFSS datasets. Inclusion criteria required valid, non-missing responses for the primary exposure (MEDCOST1) and the primary clinical outcome. Complete-case exclusions were applied to records coded as “Refused,” “Don’t know,” or “Missing.” Specifically, from the initial 1,336,111 respondents, 17,584 observations (1.32%) were excluded due to missingness in the primary exposure (n=4,808; 0.36%) or the outcome (n=13,206; 0.99%), retaining a final sample of 1,318,527 complete cases for the micro-level analysis.

Variable definitions

The primary exposure of interest was defined at the individual level via the BRFSS variable MEDCOST1 (“Was there a time in the past 12 months when you needed to see a doctor but could not because of cost?”). It is important to acknowledge that this binary metric serves as a proxy for cost-related healthcare access barriers; it does not capture the full multidimensional scope of financial toxicity, such as accumulated medical debt or psychological distress.

The primary micro-level clinical outcome was a self-reported lifetime diagnosis of angina or CHD (morbidity and survivorship), whereas the macro-level outcome evaluated state-level ischemic heart disease mortality (fatal outcomes). To quantify healthcare policy exposure at the macro level, Medicaid expansion was modeled as a continuous fractional “dose” variable ranging from 0.0 to 1.0, representing the proportion of the 36-month study period during which expansion was active. For instance, a value of 0.0 indicates no Medicaid expansion during the 2022-2024 study period, 1.0 indicates full expansion throughout the 36 months, and 0.5 indicates expansion for approximately half of the study period [12].

Statistical and spatial analysis

At the micro level, crude prevalence rates were calculated. Because age serves as a major confounder, older populations exhibit higher baseline CHD prevalence but lower financial barriers due to broad Medicare coverage, we calculated both crude and age-stratified prevalence odds ratios (PORs). To isolate the independent structural association of financial barriers, we estimated the age-adjusted prevalence odds ratio (aPOR) and its 95% CI using the Mantel-Haenszel method. Furthermore, to estimate the hypothetical public health burden on the workforce, we calculated the population attributable fraction (PAF) for the 25-44 age cohort, operating under a theoretical framework of structural association.

At the macro level, Pearson correlation coefficients (r) were initially calculated to assess bivariate ecological relationships and structural collinearity. Subsequently, a multivariable ordinary least squares (OLS) regression model was constructed to evaluate the variance in state-level CHD mortality based on age-adjusted CHD prevalence, financial toxicity, and Medicaid expansion dose.

Finally, to evaluate the geographic association and account for spatial autocorrelation, we implemented a Bayesian spatial hierarchical model using the Besag-York-Mollié (BYM) specification. A queen contiguity adjacency matrix was used to define geographic neighbors, generated from a standard U.S. Census state-level shapefile. Alaska and Hawaii were excluded from the spatially structured random effect in the BYM model due to a lack of shared contiguous borders, although they were retained in the non-spatial multivariable OLS model. Washington, DC, was included in the contiguous matrix. All spatial modeling, Markov chain Monte Carlo (MCMC) sampling, and Bayesian inferences were executed using Python (PyMC framework) [13].

Let Y_i_ represent the observed number of ischemic heart disease deaths in state *i*, and E_i_ represent the expected number of deaths based on age-standardized national rates. The relative risk (*θ*_i_) for each state was modeled assuming a Poisson distribution: *Y*_i_ 〜 Poisson (*E_i_θ_i_*)

The log-relative risk was structured as a linear combination of structural covariates and random effects:

log(*θ_i_*) = *β*_0_ + *β*_1 _(Prevalence_i_) + *β*_2_(Medcost1_i_) + *β*_3_ (Medicaid_i_) + *u*_i_ +*v*_i_ 

Where *β*_0_ is the intercept, *β*_1_ is the coefficient for age-adjusted CHD prevalence, *β*_2_ is the coefficient for state-level financial toxicity, *β*_3_ is the coefficient for Medicaid Expansion status, *u*_i_ is the spatially structured random effect, and *v*_i_ is the unstructured random effect.

Ethics statement

This study utilized publicly available, fully de-identified secondary data originally collected from human respondents via the BRFSS. Therefore, it was exempt from Institutional Review Board (IRB) review.

## Results

Individual-level and sociodemographic disparities

Our analysis revealed a classic epidemiological paradox that underscores the necessity of structural stratification. In the unadjusted model, financial barriers appeared deceptively protective against CHD (crude OR: 0.92), largely due to the overwhelming Medicare representation in the oldest cohort. However, adjusting for age via the Mantel-Haenszel method revealed the stark underlying reality: financial toxicity was associated with significantly higher odds of CHD overall (aOR: 1.80; 95% CI: 1.75-1.85).

As shown in Tables 1-2, the cardiovascular burden of financial toxicity is not distributed evenly across the population; rather, it acts as a severe structural determinant targeting historically marginalized and actively working demographics. While Medicare-eligible adults (≥65 years) experienced a moderate risk increase from cost barriers (OR=1.58; 95% CI: 1.42-1.76), the absence of healthcare affordability substantially amplified CHD prevalence in the youngest cohorts. Financial toxicity was associated with more than four-fold higher odds in young adults aged 18-24 years (OR=4.38; 95% CI: 2.76-6.93) and more than three-fold higher odds in the core workforce aged 25-44 years (OR=3.38; 95% CI: 2.78-4.12), with CHD prevalence increasing from 1,695 (0.6%) among the financially secure to 797 (2.0%) among those facing cost barriers in the latter group. Applying the population attributable fraction (PAF) as an exploratory statistical heuristic suggests that approximately 20% of the premature CHD burden in young adults aged 25-44 years could hypothetically be attributed to healthcare unaffordability. However, given the cross-sectional design, this estimate is purely theoretical and does not establish causality.

**Table 1:** Prevalence of coronary heart disease by healthcare affordability, stratified by sociodemographic characteristics (BRFSS 2022-2024). Notes: Data are derived from the pooled 2022-2024 Behavioral Risk Factor Surveillance System (BRFSS). Financial toxicity is defined as the self-reported inability to see a doctor in the past 12 months due to cost. Data are presented as unweighted absolute case counts (n), along with survey-weighted percentages and 95% CIs, in accordance with CDC complex survey design guidelines. CHD: coronary heart disease.

Demographic characteristic	Financially Secure (No Cost Barrier)		Financial Toxicity (Cost Barrier)	
	Total cohort, n (weighted %, 95% CI)	CHD cases, n (prevalence %, 95% CI)	Total cohort, n (weighted %, 95% CI)	CHD cases, n (prevalence %, 95% CI)
Overall population (N = 1,336,111)	1,213,515 (88.0%, 87.8-88.1)	71,735 (4.3%, 4.2-4.3)	117,788 (11.6%, 11.5-11.7)	6,491 (4.4%, 4.2-4.7)
Sex				
Male	576,581 (88.9%, 88.7-89.1)	42,638 (5.2%, 5.1-5.3)	51,436 (10.6%, 10.5-10.8)	3,175 (5.0%, 4.7-5.4)
Female	636,934 (87.1%, 86.9-87.2)	29,097 (3.4%, 3.3-3.4)	66,352 (12.5%, 12.3-12.7)	3,316 (3.9%, 3.6-4.3)
Age cohort				
18-24 years	71,148 (85.3%, 84.8-85.7)	198 (0.3%, 0.2-0.3)	11,422 (14.2%, 13.7-14.6)	108 (1.3%, 0.7-1.8)
25-44 years	270,088 (82.9%, 82.6-83.2)	1,695 (0.6%, 0.6-0.7)	48,420 (16.7%, 16.4-16.9)	797 (2.0%, 1.6-2.3)
45-64 years	389,718 (88.4%, 88.2-88.6)	15,711 (3.9%, 3.8-4.0)	42,901 (11.2%, 11.0-11.4)	3,143 (7.0%, 6.5-7.5)
≥65 years	482,561 (95.9%, 95.8-96.1)	54,131 (10.9%, 10.7-11.1)	15,045 (3.7%, 3.5-3.8)	2,443 (16.2%, 14.7-17.6)
Race/ethnicity				
Hispanic	108,654 (79.9%, 79.4-80.3)	3,131 (2.2%, 2.1-2.4)	25,526 (19.5%, 19.1-20.0)	876 (3.4%, 2.9-3.9)
Black, non-Hispanic	91,023 (86.4%, 86.0-86.8)	4,114 (3.5%, 3.3-3.7)	12,024 (13.2%, 12.7-13.6)	664 (4.8%, 3.7-5.8)
White, non-Hispanic	895,621 (91.1%, 91.0-91.2)	59,065 (5.2%, 5.1-5.3)	64,775 (8.6%, 8.5-8.8)	3,999 (5.0%, 4.7-5.3)
Other/multiracial	90,001 (88.0%, 87.5-88.4)	3,731 (4.1%, 4.0-4.3)	11,551 (11.3%, 10.9-11.7)	701 (6.1%, 5.6-6.5)
Educational attainment				
Elementary school (grades 1-8)	19,603 (74.0%, 72.9-75.1)	1,285 (5.2%, 4.6-5.9)	6,078 (25.1%, 24.0-26.3)	403 (6.8%, 5.1-8.6)
Grades 9-11 (some high school)	41,106 (79.3%, 78.6-80.0)	3,203 (5.6%, 5.2-6.0)	8,854 (20.0%, 19.3-20.7)	625 (5.6%, 4.7-6.5)
Grade 12 or GED (high school graduate)	294,249 (86.5%, 86.3-86.8)	18,996 (4.5%, 4.3-4.6)	35,607 (12.9%, 12.7-13.2)	1,983 (4.1%, 3.7-4.6)
College 1-3 years (some college)	319,898 (88.0%, 87.7-88.2)	21,337 (4.6%, 4.5-4.7)	34,300 (11.7%, 11.4-11.9)	1,983 (4.2%, 3.8-4.6)
College 4 years or more (graduate)	531,311 (93.3%, 93.1-93.4)	26,591 (3.4%, 3.3-3.5)	31,483 (6.5%, 6.4-6.7)	1,418 (3.3%, 2.9-3.7)
Employment status				
Employed for wages	494,732 (88.2%, 88.0-88.3)	9,805 (1.5%, 1.4-1.6)	54,112 (11.5%, 11.4-11.7)	1,346 (2.1%, 1.8-2.3)
Self-employed	102,471 (85.2%, 84.7-85.7)	3,931 (2.9%, 2.6-3.1)	12,943 (14.4%, 13.9-14.9)	400 (2.4%, 1.9-2.9)
Out of work for >1 year	19,698 (75.1%, 73.9-76.3)	893 (3.5%, 3.0-4.0)	5,413 (24.2%, 23.0-25.4)	338 (5.8%, 4.3-7.3)
Unable to work	65,087 (78.4%, 77.8-79.1)	8,316 (11.3%, 10.8-11.8)	14,267 (20.8%, 20.1-21.4)	2,029 (12.8%, 11.6-13.9)
Retired	411,335 (96.4%, 96.2-96.5)	45,526 (10.6%, 10.3-10.8)	11,466 (3.3%, 3.2-3.4)	1,787 (15.1%, 13.5-16.6)

**Table 2:** Statistical association between financial toxicity and coronary heart disease by sociodemographic cohort. Notes: ORs illustrate the comparative odds of reporting a coronary heart disease (CHD) diagnosis among individuals facing cost barriers versus those who are financially secure, stratified by demographic group. Data are derived from the 2022-2024 Behavioral Risk Factor Surveillance System (BRFSS) and adjusted for complex survey weights. *The crude OR for the overall population presents a deceptively protective association, largely due to profound age confounding, including substantial Medicare representation in the oldest cohort. The aOR, calculated using the Mantel-Haenszel method, demonstrates the underlying age-adjusted association between financial toxicity and CHD. CHD: Coronary heart disease; GED: General Educational Development; BRFSS: Behavioral Risk Factor Surveillance System; aOR: Age-adjusted odds ratio.

Demographic characteristic	Survey-weighted odds ratio (95% CI)
Overall population (N = 1,336,111)	Crude: 0.92 (0.90-0.94)*
Overall population (aOR)	aOR: 1.80 (1.75-1.85)
Sex	
Male	0.96 (0.89-1.04)
Female	1.15 (1.05-1.27)
Age cohort	
18-24 years	4.38 (2.76-6.93)
25-44 years	3.38 (2.78-4.12)
45-64 years	1.85 (1.71-2.01)
≥65 years	1.58 (1.42-1.76)
Race/ethnicity	
Hispanic	1.56 (1.32-1.85)
Black, non-Hispanic	1.39 (1.10-1.76)
White, non-Hispanic	0.96 (0.90-1.03)
Other/multiracial	1.49 (1.37-1.62)
Educational attainment	
Elementary school (grades 1-8)	1.33 (0.98-1.81)
Grades 9-11 (some high school)	1.00 (0.83-1.20)
Grade 12 or GED (high school graduate)	0.91 (0.80-1.02)
College 1-3 years (some college)	0.86 (0.82-0.90)
College 4 years or more (graduate)	0.97 (0.85-1.10)
Employment status	
Employed for wages	1.32 (1.15-1.51)
Self-employed	0.80 (0.72-0.89)
Out of work for >1 year	1.70 (1.24-2.32)
Unable to work	1.15 (1.03-1.29)
Retired	1.50 (1.33-1.70)

Racial and employment stratifications further corroborated this pattern of structural vulnerability. The inability to afford medical care significantly amplified the odds of CHD among Hispanic (OR=1.56; 95% CI: 1.32-1.85), Black, non-Hispanic (OR=1.39; 95% CI: 1.10-1.76), and Other/Multiracial (OR=1.49; 95% CI: 1.37-1.62) populations. In stark contrast, no statistically significant increase was observed within the White, non-Hispanic cohort (OR=0.96; 95% CI: 0.90-1.03) when facing similar self-reported cost barriers. Finally, prolonged systemic exclusion compounded these associations: individuals out of work for more than one year who experienced financial toxicity exhibited a CHD prevalence of 338 (5.8%), approaching the morbidity levels of elderly populations, and faced a 70% increase in the odds of chronic CHD (OR=1.70; 95% CI: 1.24-2.32) compared with their financially stable but unemployed counterparts.

Ecological correlations and structural collinearity

**Figure 1:**
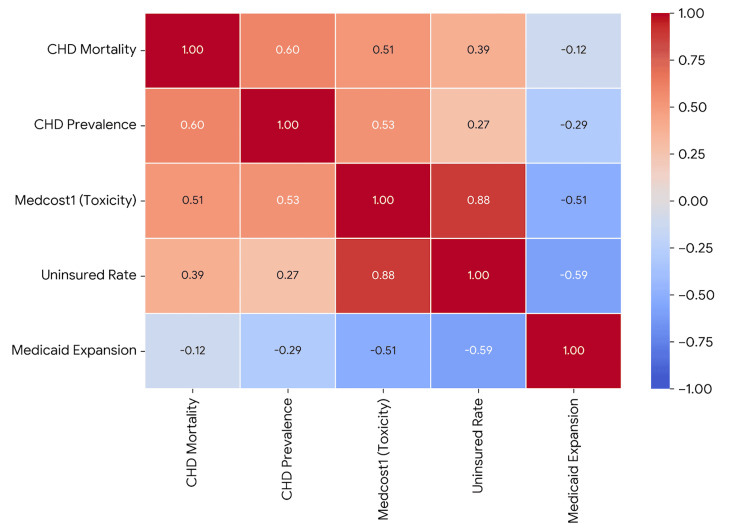
State-level ecological correlation matrix. Pearson correlation heatmap demonstrating the bivariate relationships between macro-level structural determinants and CHD outcomes. Deep red indicates strong positive correlations, while deep blue indicates strong negative correlations. All listed pairwise ecological correlations were statistically significant: uninsured rate vs. financial toxicity (r=0.88, p<0.001); CHD mortality vs. financial toxicity (r=0.51, p=0.0002); Medicaid expansion vs. financial toxicity (r=-0.51, p=0.0002); and Medicaid expansion vs. uninsured rate (r=-0.59, p<0.001). CHD: Coronary heart disease; p: p-value; r: Pearson correlation coefficient.

At the macro level (n=50 states), descriptive statistics revealed an average age-adjusted CHD mortality rate of 83.5 per 100,000 population, with substantial variation ranging from 57.9 in Colorado to 119.4 in Arkansas. Bivariate ecological analysis (Figure 1) demonstrated a very strong positive correlation between a state’s uninsured rate and its prevalence of financial toxicity (r=0.88, p<0.001), confirming that the inability to afford care is structurally dependent on lack of insurance rather than isolated individual behavior. Both of these metrics were strongly and negatively correlated with the dosage of Medicaid expansion (r=-0.59, p<0.001 and r=-0.51, p=0.0002, respectively).

Multivariable and spatial pathways to mortality

**Figure 2:**
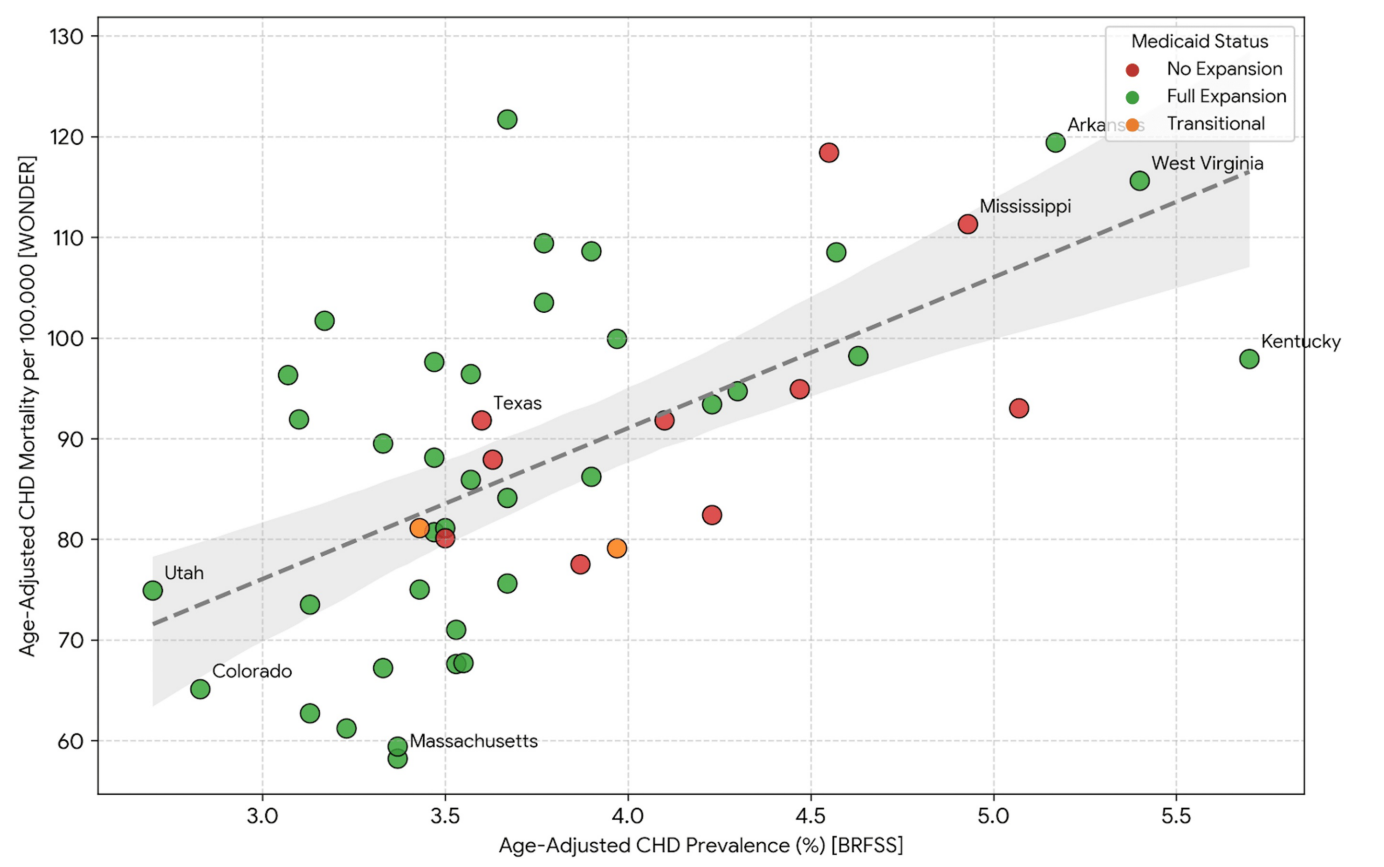
State-level CHD prevalence vs. mortality, stratified by policy. Scatter plot illustrating the positive association between age-adjusted CHD prevalence and CHD mortality per 100,000 population. Data points are color-coded by Medicaid expansion status: green, full expansion; red, no expansion; and orange, transitional. The shaded area represents the 95% CI of the regression trendline, highlighting the clustering of non-expansion states in the high-prevalence/high-mortality quadrant. CHD: Coronary heart disease.

The multivariable OLS regression model was highly significant (F(4, 45)=8.32, p<0.001), explaining 42.5% of the geographic variance in cardiovascular mortality in the United States (R²=0.425). State-level age-adjusted CHD prevalence emerged as the primary independent correlate of mortality. Controlling for policy and financial factors, every 1% absolute increase in age-adjusted CHD prevalence was associated with an excess of 16.28 cardiovascular deaths per 100,000 population (β=16.28, p<0.001; 95% CI: 9.71-22.84). As shown in Figure 2, the prevalence-mortality gradient was heavily stratified by state-level healthcare policy. States that did not expand Medicaid (red markers) clustered predominantly in the upper-right quadrant, experiencing both higher baseline prevalence of cardiovascular disease and disproportionately high mortality rates.

Transitioning to the Bayesian BYM spatial analysis confirmed financial toxicity as a credible, independent spatial correlate of cardiovascular mortality. The posterior distributions revealed a robust positive association between state-level financial barriers and CHD mortality (β=2.51, 94% highest-density interval (HDI): 0.65-4.39; representing the default highest-density interval in PyMC), independent of baseline prevalence (β=8.97, 94% HDI: 3.20-15.38). Interestingly, the direct association of Medicaid expansion displayed high variance and did not exclude zero in the spatial model (β=5.61, 94% HDI: -3.07 to 14.02).

**Figure 3:**
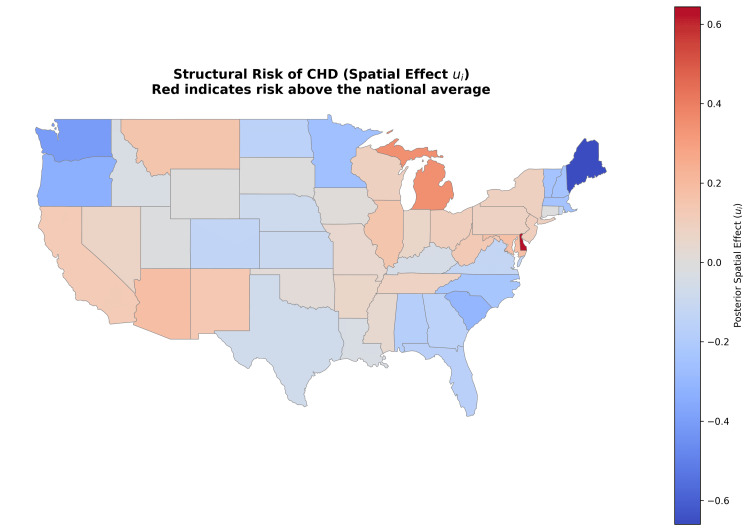
Structural risk of CHD: Bayesian spatial effect ui. Choropleth map displaying the spatially structured random effect (u_i_) extracted from the BYM hierarchical model. The color gradient reflects residual geographic risk independent of baseline CHD prevalence and modeled economic covariates. Deep red regions, indicating positive ui values, represent excess structural risk of cardiovascular mortality above the national average, demonstrating strong spatial autocorrelation in the Midwest and Mid-Atlantic regions. Conversely, blue regions, indicating negative ui values, represent a spatial protective effect, while pale or neutral colors indicate areas where mortality is adequately explained by the model’s primary covariates without additional geographic risk. BYM: Besag-York-Mollié; CHD: Coronary heart disease; uᵢ: Spatially structured random effect for state/geographic unit i.

Mapping the spatially structured random effect (ui) highlighted residual geographic clustering of cardiovascular death independent of modeled covariates. As visualized in the spatial heatmap (Figure 3), elevated residual spatial variation was concentrated in the Midwest and specific Mid-Atlantic pockets, whereas the Western states and upper Northeast maintained lower residual spatial risk. These residual regional patterns are hypothesis-generating and may reflect unmeasured structural factors, such as rural hospital closures or regional disinvestment, although these remain speculative within the scope of this model.

## Discussion

The reversal of association observed between the crude and age-adjusted estimates represents a classic example of confounding by age and underscores the importance of structural stratification when evaluating healthcare affordability and cardiovascular outcomes. In the unadjusted analysis, healthcare unaffordability appeared to be associated with slightly lower odds of CHD (OR: 0.92), an apparent paradox largely driven by the disproportionate representation of Medicare-covered older adults, among whom insurance coverage may partially mitigate the impact of healthcare costs. After age stratification and Mantel-Haenszel adjustment, the underlying association became evident, with healthcare unaffordability associated with significantly higher odds of CHD (aOR: 1.80; 95% CI: 1.75-1.85). This finding is consistent with contemporary evidence demonstrating that financial toxicity remains prevalent despite expanded insurance coverage and is increasingly driven by underinsurance, high deductibles, and rising OOP expenditures rather than lack of insurance alone [14]. Furthermore, recent Bayesian and trend analyses of BRFSS data emphasize that cost-related healthcare barriers represent deep-seated structural inequities that disproportionately affect vulnerable cohorts during periods of macroeconomic stress [15].

It is imperative to contextualize the alarming relative odds observed within the 25-44-year age demographic. Clinically, the absolute baseline prevalence of CHD in this young, active workforce is inherently and predictably low (0.6% among those without financial barriers). Therefore, the finding of 3.38-fold higher odds of CHD among young adults facing financial toxicity does not imply an absolute high-volume epidemic. Rather, it highlights a severe structural failure: financial barriers may diminish the expected biological protection of youth. By pricing young adults out of early preventive care, where modifiable risk factors such as hypertension and dyslipidemia should be managed, financial toxicity acts as a critical structural correlate of premature cardiovascular morbidity. Although causal inferences cannot be established from a cross-sectional design, applying a theoretical population attributable fraction as an exploratory statistical heuristic suggests that healthcare unaffordability may hypothetically account for approximately 20% of the observed premature CHD burden in this age group. These findings highlight the reality that financial barriers disproportionately burden working-age adults, who lack the broad coverage available to older Medicare beneficiaries while simultaneously facing competing economic demands related to employment, housing, and family responsibilities.

The macro-level consequences of these structural barriers are visually synthesized in the prevalence-mortality gradient (Figure 2). The pronounced clustering of non-Medicaid expansion states within the high-prevalence and high-mortality quadrant visually corroborates our hypothesis: legislative healthcare policy may create distinct ecosystems of cardiovascular survival. States that did not expand Medicaid not only have a higher baseline prevalence of unmanaged CHD, likely due in part to systemic financial toxicity preventing early primary care access, but also experience a disproportionate conversion of that morbidity into mortality. Crucially, while the direct association of Medicaid expansion displayed high variance in the spatial model and did not exclude zero, the visual clustering in Figure 2 underscores a vital public health reality: holding an insurance card in an expansion state does not automatically equate to functional access if regional primary care networks are saturated, yet failure to expand Medicaid may increase the likelihood of placement in the highest-risk mortality quadrant.

Beyond individual vulnerability, our Bayesian spatial analysis offers critical insights into the physical environment as a structural determinant. By mathematically controlling for baseline CHD prevalence and economic covariates, the model confirms that state-level financial toxicity is a credible, independent spatial correlate of cardiovascular death (β = 2.51, 94% HDI: 0.65-4.39). Furthermore, mapping the u_i_ from the BYM model (Figure 3) isolates a profound “geography of risk.” To clarify for clinical and public health practice, this map does not display financial toxicity itself; rather, it illustrates the residual geographic risk of cardiovascular death that remains after statistically controlling for state-level financial barriers, insurance status, and baseline CHD prevalence. Isolating this residual effect is crucial to our hypothesis because it demonstrates that financial toxicity operates alongside deeply rooted macro-social determinants, such as rural hospital closures, cardiovascular care deserts, fragmented emergency transport networks, and systemic environmental disinvestment. The concentration of residual mortality above the national average in the Midwest and Mid-Atlantic suggests that residing in a structurally compromised region may amplify cardiovascular mortality independent of an individual’s personal inability to pay. Previous ecological analyses using Bayesian frameworks have similarly demonstrated how structural socioeconomic determinants, such as regional poverty and community density, independently correlate with mortality outcomes across distinct geographic populations [16].

Crucially, our findings highlight a paradox in healthcare policy: the direct association of Medicaid expansion displayed high variance and did not exclude zero in the spatial model (β = 5.61, 94% HDI: -3.07 to 14.02). This statistical ambiguity underscores a vital public health reality: holding an insurance card does not automatically equate to having functional access to care. If regional primary care networks are saturated or low reimbursement rates disincentivize the acceptance of state-sponsored insurance, functional financial toxicity may persist for the patient.

These structural barriers cascade into well-documented clinical pathways. Our findings align with the broader framework of financial toxicity, which encompasses psychological distress, delayed care, and medication nonadherence resulting from financial strain [17,18]. Evidence from the Jackson Heart Study demonstrated that moderate-to-high financial stress was associated with a significantly increased risk of incident CHD, an association potentially mediated by interconnected behavioral, psychosocial, and metabolic pathways [19]. Chronic psychosocial stress has been linked to neuroendocrine dysregulation, systemic inflammation, endothelial dysfunction, and mechanisms involved in atherosclerotic disease progression, providing robust biological plausibility for the observed association [20].

From the perspective of family medicine, these findings suggest that affordability must be considered a clinically relevant barrier to cardiovascular prevention and disease management. The effectiveness of evidence-based therapies is substantially compromised when patients are unable to afford medications, diagnostic testing, or follow-up visits. For the primary care physician, the findings in the 25-44-year age cohort translate into a highly actionable clinical mandate: screening for financial toxicity should not be reserved solely for geriatric or visibly impoverished populations. When treating young, actively working adults presenting with modifiable risk factors such as mild hypertension or dyslipidemia, clinicians should proactively screen for cost barriers and connect these patients with financial navigation resources before these conditions progress to premature CHD [14,21,22]. At the policy and educational level, improving population-level cardiovascular outcomes requires integrating “structural competence” into primary care training, transitioning from a purely biomedical focus to a comprehensive model that actively screens for financial toxicity as a clinically relevant cardiovascular risk factor.

Limitations

Several limitations must be considered when interpreting these findings. Most importantly, the cross-sectional nature of the BRFSS individual-level data introduces temporal ambiguity between the onset of CHD and the experience of cost-related barriers to care, precluding causal inference. Reverse causation cannot be excluded; individuals living with chronic CHD may experience reduced work capacity, loss of income, or catastrophic OOP healthcare expenditures, which subsequently precipitate financial hardship. Regardless of the temporal sequence, however, the coexistence of premature cardiovascular disease and healthcare unaffordability represents a clinically critical vulnerability that demands intervention. Complete-case exclusion may also introduce selection bias, as individuals facing severe systemic barriers may be more likely to decline answering financial survey questions. Additionally, because CHD is a relatively common outcome in older cohorts, the reported PORs may overestimate the actual prevalence ratios in those specific demographic strata.

Furthermore, our macro-level spatial analysis is subject to the inherent limitations of ecological study designs. State-level aggregation of mortality, uninsurance rates, and Medicaid expansion status provides a vital macroeconomic overview but risks ecological fallacy, which is the assumption that state-level associations uniformly apply to all individuals within that state. State boundaries are political, not exact infrastructural borders. The u_i_ calculated by the BYM model likely masks significant sub-state, county-level, or ZIP code-level heterogeneity, such as the stark disparities between affluent urban centers and under-resourced rural counties within the same state.

Finally, while the Bayesian model controlled for baseline CHD prevalence and economic covariates, it could not account for all unmeasured structural confounders. Unmeasured regional variables, such as localized primary care physician shortages, rates of rural hospital closures, proximity to advanced cardiovascular care centers, and variations in state-level Medicaid reimbursement rates, likely contribute to the residual geographic variation observed in the Midwest and Mid-Atlantic. Future research should use longitudinal cohort designs and granular, county-level spatial modeling to further dissect the mechanisms linking healthcare policy, financial toxicity, and cardiovascular mortality.

## Conclusions

Financial toxicity is a profound structural correlate of cardiovascular disease, operating independently of traditional biological risk factors. Our findings demonstrate that healthcare unaffordability is associated with a significantly amplified prevalence of CHD, disproportionately burdening younger, working-age adults who often lack comprehensive safety-net coverage. Furthermore, Bayesian spatial modeling confirms that state-level financial barriers and geographic location are independent spatial correlates of cardiovascular mortality. The statistical ambiguity surrounding Medicaid expansion underscores a vital reality: providing insurance coverage is insufficient if systemic barriers to true affordability and functional access remain. Ultimately, purely biomedical interventions are fundamentally compromised when patients face severe financial distress. Addressing this crisis requires a dual approach: macro-level policies must prioritize functional healthcare affordability beyond mere insurance expansion, while primary care and family medicine training must actively integrate structural competence, financial navigation, and routine screening for financial toxicity into standard cardiovascular care.
